# Effects of smoking on the severity and transmission of pulmonary tuberculosis: A hospital-based case control study

**DOI:** 10.3389/fpubh.2023.1017967

**Published:** 2023-01-26

**Authors:** Yanmei Feng, Yue Xu, Yuan Yang, Guangzhao Yi, Huan Su, Hong Chen, Rui Guo, Jinwei Jia, Pu Wang

**Affiliations:** ^1^Department of Respiratory and Critical Care Medicine, The First Affiliated Hospital of Chongqing Medical University, Chongqing, China; ^2^Department of Infectious Disease, The First People's Hospital of Chongqing Liang Jiang New Area, Chongqing, China; ^3^Department of Cardiology, The First Affiliated Hospital of Chongqing Medical University, Chongqing, China; ^4^Department of Hospital Infection-Control, The First Affiliated Hospital of Chongqing Medical University, Chongqing, China; ^5^Department of Critical Care Medicine, The First Affiliated Hospital of Chongqing Medical University, Chongqing, China; ^6^Department of Respiratory Medicine, Chongqing Emergency Medical Center, Chongqing University Central Hospital, Chongqing, China

**Keywords:** pulmonary tuberculosis, smoking, severity of the disease, positive tubercle bacilli, transmission

## Abstract

**Introduction:**

There is a high incidence of both smoking and tuberculosis (TB) in China. This study examined the risk factors for severe pulmonary TB (PTB) and positive tubercle bacilli in sputum.

**Methods:**

We conducted a retrospective case-control study in a tertiary hospital from January 2017 to December 2018 (*n* = 917). The clinical and biological characteristics of patients were collected, and univariable and multivariable logistic regression analyses were performed to assess the factors associated with smoking in terms of the severity and transmission of PTB.

**Results:**

Positive tubercle bacilli in sputum and severe PTB were much higher in smoking patients. Together with nutrition status, heavy smoking exhibited a 284% greater risk in severe PTB. Positive tubercle bacilli in sputum was significantly associated with hypoproteinemia and smoking regardless of the status, duration, and degree.

**Conclusion:**

Because cigarette smoking was strongly and inversely associated with hypoproteinemia, we conclude that smoking plays a critical role in the severity and transmission of PTB. Smoking cessation interventions should be employed to prevent severe PTB and decrease the transmission of PTB.

## Background

Although efforts are underway worldwide to end the global tuberculosis (TB) epidemic, TB remains a leading cause of infectious disease, creating a significant public health burden worldwide. As reported by WHO, in 2021, an estimated 10.6 million people were infected with TB, and 1.6 million people died from it (1). Currently, evidence of the impact of smoking on TB is growing (2–4). China, which has a high TB burden, also ranks among the top countries in the world in the number of smokers, particularly among adolescents (5). It has been estimated that if 80% coverage by the WHO-recommended strategy for TB control (directly observed treatment, short-course chemotherapy, or DOTS) is sustained, complete smoking cessation and complete elimination of solid fuel use by 2033 would reduce the projected annual TB incidence by 14–52% (6). Therefore, to achieve the goal of ending TB by 2035, it is important to understand the characteristics of smoking-related TB. Previous studies of smoking-related TB have largely focused on the risk of developing TB, treatment outcomes, and prognoses (2, 7). Amere et al. (2) estimated the incidence and mortality of TB among smoking patients in 32 high-TB-burden countries, reporting 17.6% (95%CI = 8.4–21.4) TB cases and 15.2% (95%CI = 15.9–37.6) TB mortality attributable to smoking. Burusie et al. (8) systematically reviewed 22 studies and found that smoking significantly increased the likelihood of poor TB outcomes by 51% (OR = 1.51, 95%CI = 1.3–1.70, I-square = 75.1%). These findings are notable, but to the best of our knowledge, there is little information available about the severity and transmission of the disease in smoking patients with PTB, particularly in China.

This study investigated the severity of the disease and level of disease transmission for PTB in smoking patients. Furthermore, to explore the risk factors for severe PTB and PTB transmission, we analyzed smoking and associated factors in terms of the degree, duration, and status of smoking.

## Methods

### Study design and populations

**Figure 1:**
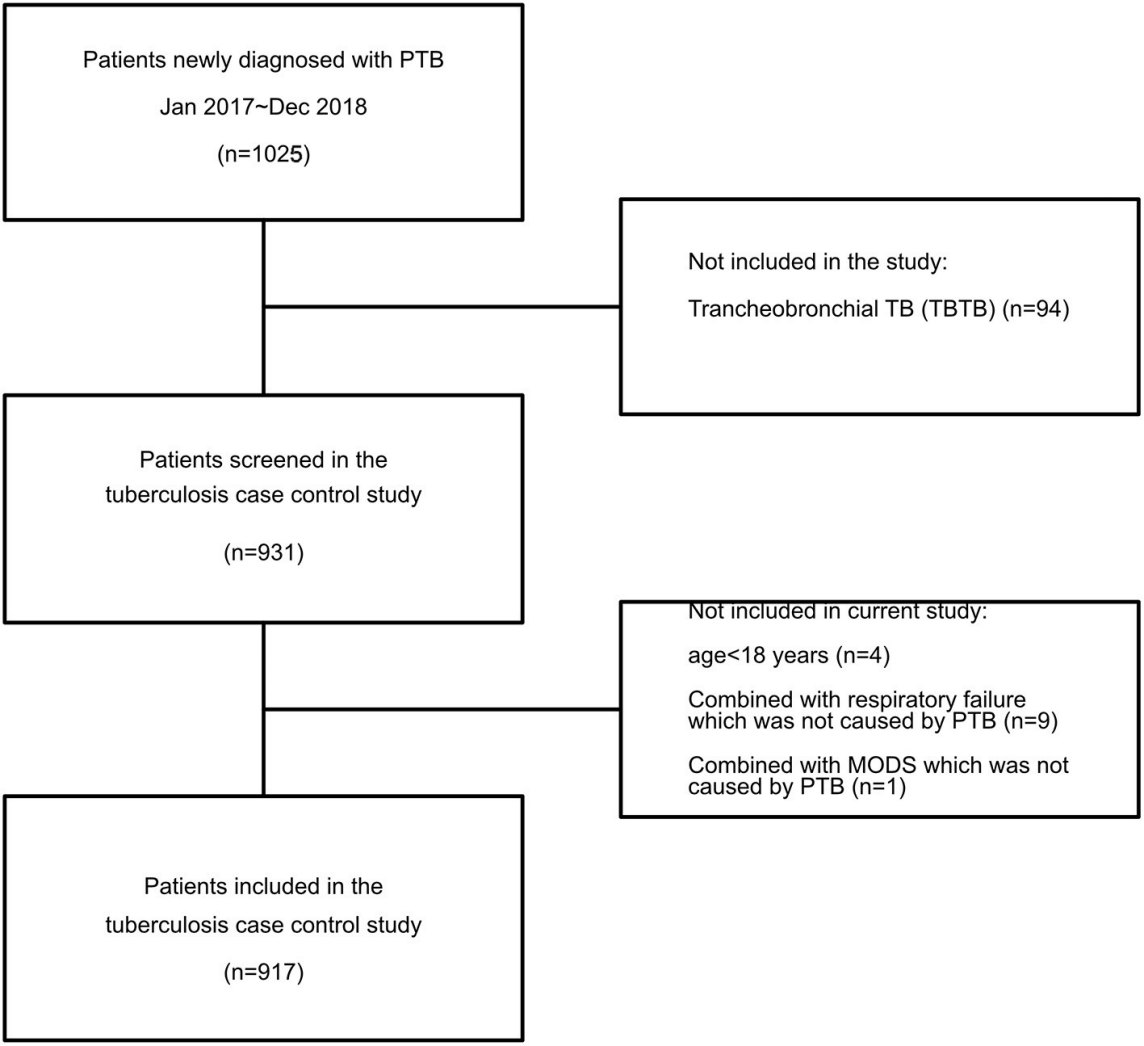
Flow diagram.

A case-control study was conducted among patients with PTB at a tertiary hospital between January 2017 and December 2018 in southwest China. All patients over the age of 18 who visited our hospital with a diagnosis of PTB were invited to participate. Those who experienced multiple organ dysfunction syndrome (MODS), septic shock, or respiratory failure not caused by PTB were excluded; in addition, those who were unable to obtain intact information data were also excluded (Figure 1). Data on all of the enrolled patients were collected using the hospital's medical record systems and telephone interviews. The survey was designed to investigate smoking among patients with PTB as well as the risk factors for the severity and transmission of PTB. No personally identifying details were collected. Consent was obtained during the telephone interviews, and only participants who provided consent entered the survey.

### Data collection, definitions, and criteria

Demographic information, clinical data, and self-reported cigarette smoking status were collected using the medical records system and telephone interviews. Smokers were defined as individuals who smoked regularly or had done so for at least 6 months during their lives. Non-smokers were defined as patients who neither smoked more than 100 cigarettes in their lifetime nor were currently smoking. Smokers were divided into current smokers and ex-smokers (past smokers). To prevent misclassification of patients who temporarily quit smoking at the onset of symptoms, the definition of current smoker was expanded to include past smokers who had stopped smoking after the onset of PTB-associated symptoms. To further clarify the influence of smoking on PTB, the extent of smoking was evaluated. Heavy smoking and light smoking were defined following a previous study (9) such that heavy smoking was 20 or more cigarettes per day, or more than 20 pack-years, and light smoking was <20 cigarettes per day, or <20 pack-years.

The Bandim TBscore is an easily implemented self-rated instrument for evaluating severity in patients with PTB (10). It assesses five symptoms (cough, hemoptysis, dyspnea, chest pain, and night sweats) and six signs [pale inferior conjunctivae, pulse >90 per minute, positive findings at lung auscultation, temperature >37°C (axillary), body mass index (BMI) <18/ <16, and mid-upper-arm circumference (MUAC) <220 mm/ <200 mm]. Each variable contributes one point. BMI and MUAC contribute an extra point each, if BMI <16 and MUAC <200 mm; hence, the maximum score is 13 (Table 1). A score of 8 or greater is defined as severe TB, which is associated with a strong prognostic capacity of mortality. As MUAC data could not be obtained, we adjusted the self-rated health score to assess disease severity. As in a previous study (11), because hypoproteinemia and bilateral lung involvement have been shown to be associated with increased mortality in PTB (12, 13), we adjusted the Bandim TBscore system for hypoproteinemia, with bilateral lung involvement in CT or X-ray instead of MUAC (Table 1).

**Table 1:** TBscore and adjusted TBscore.

**Variables**	**TBscore**	**Adjusted TBscore**
**Symptoms**		
Cough	1	1
Dyspnea	1	1
Chest pain	1	1
Night sweats	1	1
**Signs**		
Anemia	1	1
Pulse >90 beats/min	1	1
Positive finding at lung auscultation	1	1
Temperature >37°C	1	1
BMI <18	1	1
BMI <16	1	1
MUAC <220 mm	1	–
MUAC <200 mm	1	–
Hypoproteinemia	–	1
Bilateral lung involvement	–	1
**Total**	13	13

BMI, body mass index; MUAC, mid upper arm circumference.

### Statistical analysis

All data were analyzed using R software version 3.6.2 (R Foundation for Statistical Computing). Quantitative variables were expressed as means/medians and standard deviations. Qualitative variables were summarized as counts and proportions for each category. Smokers' and non-smokers' characteristics were compared using Pearson's χ^2^ test. To further evaluate the factors (mainly focused on smoking) that influence the severity of PTB and positive tubercle bacilli in sputum, univariable and multi-variable logistic regression analyses were conducted. The variables for inclusion were carefully chosen, using statistically significant associations in univariate analysis. The results are presented as *P*-values, odds ratios (ORs), and 95% confidence interval (CIs). A *P*-value of < 0.05 was considered statistically significant.

### Ethical considerations

This study adhered to the tenets of the Declaration of Helsinki, and the ethics committee of the First Affiliated Hospital of Chongqing Medical University approved the study (No. 2020-140). All patient records were anonymized before analysis. All subjects received electronic information before inclusion and provided oral consent in the telephone interview.

## Results

### Characteristics of participants

In all, 931 inpatients at the First Affiliated Hospital of Chongqing Medical University suffering from PTB (between January 2017 and December 2018) were screened, of which 14 were excluded because of age younger than 18 years or having either MODS or dysfunction respiratory failure not caused by PTB. As shown in Table 2, there were 447 smokers among the enrolled patients, of whom 98.2% were male. The mean age of smokers was much higher than that of the non-smokers (58.1 ± 15.4 vs. 45.2 ± 19.9, *P* < 0.001). In 64.2% of the smokers, smoking duration was more than 20 years, and 62.2% of smokers had not given up smoking (Supplementary Table 1). Although the BMI was similar between smokers and non-smokers (20.6 ± 3.05 vs. 20.7 ± 3.0), the proportion of hypoproteinemia was much higher in the smoking PTB patients (*P* < 0.001). In addition to the higher positive tubercle bacilli in smokers, the smoking patients also had a higher disease severity (TBscore >8) and a higher Charlson comorbidity index (CCI). However, we did not find a significant difference in multi-system TB between smokers and non-smokers.

**Table 2:** Characteristics of patients and factors related to smoking at the time of TB diagnosis.

	**Non-smoking** **(*N* = 470)**	**Smoking** **(*N* = 447)**	***P*** **-value**
**Sex**			
Female	319 (67.9%)	8 (1.8%)	< 0.001
Male	151 (32.1%)	439 (98.2%)	
**Age (years)**			
Mean (SD)	45.2 (19.9)	58.1 (15.4)	< 0.001
**BMI (kg/m** ^2^ **)**			
Mean (SD)	20.6 (3.05)	20.7 (15.4)	0.658
**Hypoproteinemia**			
No	321 (68.3%)	231 (51.7%)	< 0.001
Yes	149 (31.7%)	216 (48.5%)	
**BTS**			
Mean (SD)	4.26 (1.91)	4.91 (1.96)	< 0.001
**Multi-system TB**			
No	437 (93%)	418 (93.5%)	0.849
Yes	33 (7%)	29 (6.5%)	
**Tubercle bacilli**			
Negative	290 (61.7%)	203 (45.4%)	< 0.001
Positive	180 (38.3%)	244 (54.6%)	
**BTS level**			
< 8	446 (94.9%)	403 (90.2%)	0.009.4
≥8	24 (5.6%)	44 (9.8%)	
**CCI**			
0	338 (71.9%)	229 (51.2%)	< 0.001
1	94 (20%)	157 (35.1%)	
2	30 (6.4%)	39 (8.7%)	
≥3	8 (1.7%)	22 (4.9%)	

BMI, body mass index; TB, tuberculosis; BTS, Bandim TBscore; CCI, Charlson comorbidity index.

### Smoking and associated factors in severe PTB

Table 3 presents the ORs for severe PTB. The risk factors were age, sex, BMI, hypoproteinemia, smoking, and CCI. To further explore the influence of smoking on severe PTB, the smokers were classified into different types based on smoking status, degree, and duration. As the *P*-value of those variables was more than 0.05 in univariable analysis, the analyses were adjusted for the factors that did not enter the model. Age more than 70 years, BMI in the underweight group, and hypoproteinemia were associated with severe PTB. Although smoking status and smoking duration did not exhibit a relationship with severe PTB, heavy smokers exhibited an increased rate of severe PTB, 284% of that for other patients (AOR = 3.84; 95% CI = 1.45–10.2, *P* = 0.007).

**Table 3:** Odds ratios (ORs) for severe PTB in smoking and associated factors.

**Variables**	**Univariable**		**Multivariable**	
	**OR (95% CI)**	***P*** **-value**	**AOR (95% CI)**	***P*** **-value**
**Age (years)**				
18–39	Reference			
40–59	0.78 (0.4–1.53)	0.467	0.62 (0.28–1.42)	0.261
60–69	1.67 (0.88–3.18)	0.118	0.93 (0.42–2.07)	0.862
>70	0.88 (0.4–1.95)	0.761	0.31 (0.12–0.79)	0.014
**Sex**				
Female	Reference			
Male	1.73 (0.98–3.04)	0.059		
**BMI**				
Normal	Reference			
Underweight	6.32 (3.63–11)	< 0.001	5.81 (3.17–10.63)	< 0.001
Overweight	0.81 (0.24–2.77)	0.736	1.13 (0.31–4.07)	0.856
Obese	0.99 (0.29–3.41)	0.99	1.24 (0.34–4.52)	0.744
**Hypoproteinemia**				
No	Reference			
Yes	18.62 (7.96–43.55)	< 0.001	17.27 (7.17–41.62)	< 0.001
**CCI**				
0	Reference			
1	0.94 (0.53–1.67)	0.836		
2	0.75 (0.26–2.16)	0.593		
≥3	1.35 (0.39–4.64)	0.63		
**Smoking status**				
Ex-smoker	Reference			
Current smoker	2.03 (1.21–3.40)	0.007	2.4 (1–5.8)	0.051
**Smoking degree**				
Non-smoker	Reference			
Light smoker	1.41 (0.75–2.64)	0.29	1.66 (0.63–4.36)	0.306
Heavy smoker	2.93 (1.63–5.24)	< 0.001	3.84 (1.45–10.2)	0.007
**Smoking duration**				
Non-smoker	Reference			
< 10 years	2.44 (1.05–5.67)	0.039	2.86 (0.92–8.9)	0.071
10–20 years	1.08 (0.40–2.91)	0.878	1.49 (0.42–5.28)	0.536
>20 years	2.25 (1.29–3.92)	0.004	2.6 (0.99–6.84)	0.052

BMI, body mass index; PTB, pulmonary tuberculosis; OR, odds ratios; AOR, adjusted odds ratios; CCI, Charlson comorbidity index. Except for independent variables, the AOR for multivariable analyses was adjusted for factors that did not enter the model.

### Smoking and associated factors in positive tubercle bacilli

The transmission of PTB was evaluated using positive tubercle bacilli. According to multivariable analysis (Table 4), the following factors were independently associated with PTB transmission: BMI in the underweight group (AOR = 1.58; 95% CI = 1.12–2.22), hypoproteinemia (AOR = 1.67; 95%CI = 1.25–2.22), CCI with 1 score (AOR = 1.79; 95% CI = 1.3–2.47) and more than 3 scores (AOR = 4.28, 95%CI = 1.77–10.35), smoking status in current smokers (AOR = 1.64; 95% CI = 1.11–2.42), smoking degree in heavy smokers (AOR = 2.11; 95% CI = 1.33–3.33), and smoking duration of more than 20 years (AOR = 1.82; 95% CI = 1.18–2.82) or < 10 years (AOR = 1.85; 95% CI = 1.03–3.3).

**Table 4:** Odds ratios (ORs) for transmission of PTB in smoking and associated factors.

**Variables**	**Univariable**		**Multivariable**	
	**OR (95% CI)**	***P*** **-value**	**AOR (95% CI)**	***P*** **-value**
**Age (years)**				
18–39	Reference			
40–59	1.24 (0.89–1.73)	0.212	1.01 (0.7–1.45)	0.964
60–69	1.96 (1.34–2.85)	< 0.001	1.39 (0.92–2.11)	0.115
>70	1.9 (1.27–2.83)	0.002	1.22 (0.78–1.9)	0.384
**Sex**				
Female	Reference			
Male	1.63 (1.24–2.14)	< 0.001	1.34 (1–1.8)	0.05
**BMI**				
Normal	Reference			
Underweight	1.68 (1.21–2.32)	0.002	1.58 (1.12–2.22)	0.009
Overweight	0.96 (0.62–1.49)	0.865	0.97 (0.61–1.54)	0.909
Obese	0.75 (0.46–1.22)	0.25	0.68 (0.41–1.14)	0.144
**Hypoproteinemia**				
No	Reference			
Yes	2.1 (1.6–2.75)	< 0.001	1.67 (1.25–2.22)	< 0.001
**CCI**				
0	Reference			
1	1.98 (1.47–2.68)	< 0.001	1.79 (1.3–2.47)	< 0.001
2	1.22 (0.74–2.02)	0.435	1.06 (0.62–1.68)	0.838
≥3	4.92 (2.08–11.66)	< 0.001	4.28 (1.8–10.35)	0.001
**Smoking status**				
Ex-smoker	Reference			
Current smoker	1.94 (1.49–2.52)	< 0.001	1.64 (1.11–2.42)	0.013
**Smoking degree**				
Non-smoker	Reference			
Light smoker	1.64 (1.2–2.23)	0.002	1.39 (0.92–2.12)	0.122
Heavy smoker	2.44 (1.73–3.44)	< 0.001	2.11 (1.33–3.33)	0.001
**Smoking duration**				
Non-smoker	Reference			
< 10 years	1.86 (1.12–3.1)	0.016	1.85 (1.03–3.3)	0.039
10–20 years	1.21 (0.77–1.9)	0.415	1.11 (0.64–1.92)	0.715
> 20 years	2.27 (1.69–3.07)	< 0.001	1.82 (1.18–2.82)	0.007

BMI, body mass index; PTB, pulmonary tuberculosis; OR, odds ratios; AOR, adjusted odds ratios; CCI, Charlson comorbidity index; adjusted for age and sex. Except for the independent variable, the AOR or multivariable analyses was adjusted for factors that did not enter the model.

## Discussion

PTB remains the respiratory infectious disease with the highest mortality rate worldwide. Countermeasures are needed to control the source of infection and reduce mortality. It is very important to know the risk factors and intervene in advance. In this study, we found that smoking and nutrition status were independent risk factors for severe PTB and bacterial-positive TB.

In line with a previous study (13), more than 95% (439/447) of smokers were male in our study, whereas only 2% (8/325) of females were smokers. The sex difference in smoking is consistent with smoking prevalence in China (5, 14): men must stop using tobacco.

Hypoproteinemia is an independent risk factor for various diseases (15–17). Although evidence already exists for its effect on treatment outcomes (18), we further evaluated the relationship between hypoproteinemia and PTB in terms of severity and positive tubercle bacilli. Hypoproteinemia was not only associated with positive tubercle bacilli but could also be seen as an independent risk factor for severe PTB. Previous studies have shown that cigarette smoking is strongly and inversely associated with serum concentrations of albumin (19). We observed the same phenomenon in our study, with a higher proportion of hypoproteinemia in patients with a smoking history. Conducting tobacco cessation education should be considered equally as important as nutritional support.

Severe PTB is associated with higher mortality. It is essential to explore risk factors for critically ill patients. However, no diagnostic criteria have been provided for severe PTB. The Bandim TBscore system, first developed by Wejse in 2009, is an effective and convenient scoring instrument for the assessment of PTB disease severity. Thus, we evaluated the severity of PTB with an adjusted Bandim TBscore and found that the proportion of severe PTB was higher in smokers. By further evaluating the influence of smoking status, degree, and duration in regard to PTB severity, we found that heavy smoking was an independent factor for severe PTB. This result is in concordance with a previous study (2), in particular, that smoking contributes to PTB incidence and mortality in high-TB-burden countries. This provided a hint that to decrease the incidence of severe PTB, smoking, particularly heavy smoking, should be avoided.

TB is an infectious disease that claims many human lives. Preventing person-to-person spread is central to halting the TB epidemic. The transmission of TB requires the expulsion of viable tubercle bacilli from an active source case (20), so it is critical to explore the risk factors for positive tubercle bacilli in sputum. It has been reported that higher sputum mycobacterial loads are found in smoking patients (21). This was the cased in our study. Among other risk factors, we found that hypoproteinemia, BMI at underweight level, CCI, and smoking were strongly associated with positive tubercle bacilli in sputum. This connection varies with status, degree, and duration of smoking. As smoking is a risk factor for comorbidites (e.g., chronic pulmonary disease, heart disease, diabetes et al.) and abnormal nutrition status (e.g., hypoproteinemia) (22–25), we conclude that smoking plays a key role in positive tubercle bacilli in sputum.

Previous studies have also reported the negative influence of comorbidities on clinical outcomes in PTB (26). The CCI was developed as a comorbidity-based indicator for estimating the risk of death and classifying the severity of a patient's clinical condition (27). A previous study reported that a CCI score >3 was an independent risk factor for all-cause death in PTB patients (28). Min et al. (29) also found that LIBT patients with a CCI score of 3 or higher were less likely to complete its treatment, indicating a poorer prognosis. However, the role of CCI in the severity and spread of TB has not been established. In this study, we investigated the use of this scoring system in evaluating the severity and transmission of PTB, with a particular focus on smoking populations. The results revealed that the CCI score was significantly higher in the smoking group than in the non-smoking group (*P* < 0.001). According to further evaluation with multivariable logistic analysis, CCI was not associated with the severity of PTB, while there was a relationship between CCI and positive TB in sputum. Because the sample size with a CCI score of more than 2 was small in this study, more evidence is needed to confirm the link between CCI and the disease severity/transmission of PTB.

This study had some limitations, such as the retrospective data collection. Due to the properties of the case-control study, we were unable to identify the timepoint for mycobacteria in sputum conversion to negative and the prognosis of PTB smokers. In addition, the sample size, particularly for smoking subgroups such as status, duration, and degree, was insufficient. To further clarify the important role that smoking plays in PTB, the sample size should be enlarged. Moreover, we confined our study to a single center. The results may be affected by the nature and policies of our hospital. To understand the role of various states of smoking in PTB development, the logical next step would be to perform a study of this type in Chongqing or the southwestern region of China in a multicenter prospective study.

## Conclusion

Our findings indicate risk factors for severe PTB and PTB transmission. Smoking plays a critical role in the severity and transmission of PTB. Smoking cessation interventions should be offered to PTB patients. Further trials are needed to investigate the possible mechanisms and possible benefits of early smoking cessation in the improvement of the clinical prognosis of PTB patients.

## Data availability statement

The original contributions presented in the study are included in the article/Supplementary material, further inquiries can be directed to the corresponding authors.

## Ethics statement

The studies involving human participants were reviewed and approved by committee of the First Affiliated Hospital of Chongqing Medical University (No. 2020-140). The patients/participants provided their written informed consent to participate in this study.

## Author contributions

YF analyzed all of the data and wrote the manuscript. YF, HC, and YX reviewed the literature. HS, YX, and GY collected the raw data. YY assisted in the statistical analysis. RG, JJ, and PW edited and supervised the writing of the manuscript. All authors contributed to the article and approved the submitted version.
