# Supplementary material for: Effects of smoking on the severity and transmission of pulmonary tuberculosis: A hospital-based case control study
Source: Front Public Health. 2023 Jan 26;11:1017967. doi: 10.3389/fpubh.2023.1017967 (PMC9909179; doi:10.3389/fpubh.2023.1017967)
Supplement: Supplementary file 1 [file Table_1.DOCX]

**Table 1.** The smoking features of PTB patients

| **Character** | **Number** | **Ratio** |
| --- | --- | --- |
| **Smoking history** |  |  |
| Never smoker | 470 | 51.3% (470/917) |
| smoker | 447 | 48.7% (447/917) |
| **Smoking intensity** |  |  |
| Heavy smokers | 190 | 42.5% (190/447) |
| Light smokers | 257 | 57.5% (257/447) |
| **Smoking duration** |  |  |
| <10 years | 69 | 15.4% (69/447) |
| 10~20 years | 91 | 20.4% (91/447) |
| >20 years | 287 | 64.2% (287/447) |
| **Smoking status** |  |  |
| Current smokers | 278 | 62.2% (287/447) |
| Ex-smokers |  |  |
| >1 years | 127 | 28.4% (127/447) |
| <1 year | 42 | 9.4% (42/447) |
